# Lactate Beyond a Waste Metabolite: Metabolic Affairs and Signaling in Malignancy

**DOI:** 10.3389/fonc.2020.00231

**Published:** 2020-03-18

**Authors:** Fátima Baltazar, Julieta Afonso, Marta Costa, Sara Granja

**Affiliations:** ^1^School of Medicine, Life and Health Sciences Research Institute (ICVS), University of Minho, Braga, Portugal; ^2^ICVS/3B's—PT Government Associate Laboratory, Guimarães, Portugal

**Keywords:** lactate, warburg effect, monocarboxylate transporters, GPR81, metabolic fuel, lactate shuttles, signaling molecule

## Abstract

To sustain their high proliferation rates, most cancer cells rely on glycolytic metabolism, with production of lactic acid. For many years, lactate was seen as a metabolic waste of glycolytic metabolism; however, recent evidence has revealed new roles of lactate in the tumor microenvironment, either as metabolic fuel or as a signaling molecule. Lactate plays a key role in the different models of metabolic crosstalk proposed in malignant tumors: among cancer cells displaying complementary metabolic phenotypes and between cancer cells and other tumor microenvironment associated cells, including endothelial cells, fibroblasts, and diverse immune cells. This cell metabolic symbiosis/slavery supports several cancer aggressiveness features, including increased angiogenesis, immunological escape, invasion, metastasis, and resistance to therapy. Lactate transport is mediated by the monocarboxylate transporter (MCT) family, while another large family of G protein-coupled receptors (GPCRs), not yet fully characterized in the cancer context, is involved in lactate/acidosis signaling. In this mini-review, we will focus on the role of lactate in the tumor microenvironment, from metabolic affairs to signaling, including the function of lactate in the cancer–cancer and cancer–stromal shuttles, as well as a signaling oncometabolite. We will also review the prognostic value of lactate metabolism and therapeutic approaches designed to target lactate production and transport.

## Introduction

The first discoveries involving lactate were reported in 1808, when it was described in the muscle of animals; only many years later was lactate associated with energy metabolism in muscle contraction (1, 2). The glycolysis pathway, transformation of glucose into pyruvate and ATP, was described in the 1940s by the joined efforts of numerous scientists, in a cascade-like chronology. It started with the discovery of fermentation in microorganisms by Louis Pasteur; then, Meyerhof et al. described the lactate cycle, providing essential knowledge on the transformation of energy in cells (3–5).

Lactate formation and functions were incorrectly described for a long time, since lactate was considered as a waste product of cellular metabolism (6). Although history did not give lactate its due importance, it is believed at presentthat lactate has a crucial role, especially as a shuttle molecule. The concept was introduced by Brooks more than 30 years ago (7–9), and despite some initial disbelief (10, 11), several reports have finally acknowledged the role of lactate in shuttles between glycolytic and oxidative cells, being the product of one and used by another (12). It is well-established that lactate is formed from the reduction of pyruvate via lactate dehydrogenase (LDHA), under aerobic or anaerobic conditions, produced, and transformed continuously by resting/exercising muscle, brain, heart, and gut tissues (13). Lactate is a major source of energy, the major gluconeogenic precursor and, as a signaling molecule, is capable of inducing autocrine, paracrine, and endocrine-like effects. This molecule is responsible for several homeostatic functions: For instance, in hepatocytes, it feeds gluconeogenesis; in the brain, it is used by astrocytes and neurons for oxidative metabolism (12, 14).

Lactate homeostasis in a healthy environment requires adequate transporters. The lactate transporters, monocarboxylate transporters (MCTs), are members of the SLC16 gene family and several have been identified by gene homology, as it will be further explained (15). Physiological levels of lactate are considered to be in the range of 1.5–3 mM in blood and tissue from healthy individuals (14, 16); higher values are usually an indication of a health problem. Lactate shuttles are key players in many conditions involving pregnancy and reproduction (17, 18), the human heart (19), brain (12), and cancer (13). The use of lactate levels as a marker of clinical outcome was first suggested in 1964 by Broder and Weil, when studying patients with undifferentiated shock (20). Since then, high lactate levels have been associated with several diseases such as shock, cardiac arrest, trauma, ischemia, diabetic ketoacidosis, liver dysfunction, and sepsis (21). Lactate also modulates the immune system and promotes immune-inflammatory responses (22). The levels of lactate are increased in several inflammatory and autoimmune disorders, and lactate transporters were overexpressed at the surface of immune cells (14, 23). Lactate accumulation and transport has become particularly relevant in rheumatoid arthritis, where MCT4 inhibition was pointed as a possible therapeutic strategy (24). In the cancer setting, Otto Warburg was the first to observe that tumor cells share a common metabolic feature: high glucose consumption and increased glycolysis leading to lactate production, regardless of oxygen availability (25, 26).

## Role of Lactate in Cancer Metabolic Rewiring

Cancer metabolism emerged as an area of research that has increasingly gained attention in the last decades. In order to sustain the proliferative phenotype, cancer cells enroll metabolic changes, such as the “Warburg effect” disclosed by Otto Warburg in 1926 (27). These changes consist on upregulation of glucose metabolism (glycolysis) even in the presence of oxygen, thereby producing high levels of lactate and reducing the use of the tricarboxylic acid (TCA) cycle. This addictive glycolytic phenotype arises as a distinctive metabolic characteristic of many types of cancer, being introduced as a new hallmark of cancer in 2011 (28).

### Oncogenic Triggers of Glycolytic Metabolism

Tumorigenesis is characterized by genetic alterations, and several findings demonstrate that high expression of specific transcription factors or oncogenic tumor pathways, principally MYC, hypoxia-inducible factor-1 alpha (HIF-1α), nuclear factor kappa-light-chain-enhancer of activated B cells (NF-κB), and phosphatidylinositol-3-OH kinase (PI3K), can sustain the Warburg effect. As the tumor starts to grow, oxygen diffusion becomes limited and cancer cells respond to these environmental changes by upregulating HIF-1α (29). HIF-1α, in turn, induces the overexpression of key players in the conversion of glucose into lactate, such as glucose transporters (GLUTs) and hexokinase (HK) 1 and 2, which are responsible for the initial steps of glycolysis; lactate dehydrogenase A (LDHA), which transforms pyruvate into lactate (30, 31); and the lactate-extruding monocarboxylate transporter 4 (MCT4) (32). Conversely, HIF-1α blocks the entry of pyruvate into the TCA cycle by upregulating pyruvate dehydrogenase kinase 1 (PDK1), driving tumor cell energy to glycolysis (33). The major oncogene *Ras*, when mutated, can also induce glycolysis through the activation of the mammalian target of rapamycin complex I (mTORC1). Akt kinase activation by PI3K results in increased glucose uptake, HK2 targeting to the mitochondria, and increase in glycolytic flux (34), while the transcription factor MYC increases glutaminolysis and upregulates MCT1 expression (35). Cancer cell metabolism is also influenced by the activity of tumor suppressor genes. Loss of the p53 protein prevents expression of the synthesis of cytochrome c oxidase (*SCO2*) gene, decreasing mitochondrial respiration (36). Lactate can also function as a paracrine tumor molecule (37). Acidosis often precedes angiogenesis and lactate can stimulate HIF expression independently of hypoxia (38). Thus, instead of one event promoting the Warburg effect, numerous factors play a role in determining the fate of glucose in cancer cells. Also true is that somatic mutations in genes involved in metabolism either cause/predispose cells to become malignant. For instance, mutations in succinate dehydrogenase are related to paraganglioma, and mutations in fumarase can induce leiomyoma and leiomyosarcoma formation (39); isocitrate dehydrogenase mutations are related to glioma development (39).

### Lactate Transport in Cancer

The major oncometabolite resulting from tumor metabolic rewiring is lactate, which is abundant in the tumor microenvironment (TME). Because lactic acid is hydrophilic and a weak acid, its transport across membranes requires transporters that belong to the monocarboxylate transporter family. MCTs 1–4 facilitate the transmembrane H^+^-linked transport of monocarboxylates, including lactate, pyruvate, acetoacetate, and β-hydroxybutyrate (15), having the cell surface glycoprotein CD147 as an obligatory chaperone (40). MCT1 and MCT4 isoforms are strongly associated with the hyperglycolytic phenotype of cancer cells. These transporters display distinct affinities for monocarboxylic acids that are associated with their expression patterns within tissues (41). MCT1 expression was identified in most tissues, being associated with the uptake/extrusion of lactate, while MCT4 has an important role in the export of lactate in highly glycolytic tissues. MCTs have been widely studied by our group and others, and found to be robustly expressed in a variety of solid human tumors such as colon, glioblastoma, breast, prostate, stomach and others, as depicted in Table 1 [for a comprehensive review see (41, 72)]. MCT expression/cell localization can differ from cancer to cancer. Importantly, the prognostic potential of MCTs was found in various tumor types (Table 1). Given the key role of MCTs in cancer, these transporters are promising therapeutic targets in cancer. A less studied family of lactate transporters, also known to facilitate the transport of monocarboxylates, are the sodium-coupled monocarboxylate transporters (SMCTs), containing two members, SLC5A8 and SLC5A12 (73).

**Table 1:** Expression pattern and prognostic value of MCT1 and MCT4 in human cancer.

**Tumor type**	**Expression**	**Prognostic value**
Brain	↑ MCT1 (42) ↑ MCT4 (42)	
Head and Neck	↑ MCT1 (43) ↑ MCT4 (43)	MCT4 expression associated with advanced TNM stage (43)
Breast	↑ MCT1 (44) ↑ MCT4 (44)	MCT1/CD147 expression associated with basal-like subtype advanced TNM stage (44) MCT4 expression identified as an independent prognostic factor for MFS and OS (45)
Lung	↑ MCT1 (46) ↑ MCT4 (46)	MCT1 low expression associated with shorter DFS (47) MCT4 expression associated with shorter OS (48) and DFS (47)
Liver	↓MCT1 (49) ↑ MCT4 (49)	MCT4 expression identified as an independent prognostic factor for DFS and OS (50)
Pancreas	↑MCT1 (51) ↑ MCT4 (51)	MCT1 expression associated with extended OS and PFS and decreased nodal metastasis (52) MCT4 expression in CAFs associated with shorter OS (52)
Stomach	↓MCT1 (53) ↑ MCT4 (53)	
Colorectal	↑MCT1 (54) ↑ MCT4 (54)	MCT4 expression associated with metastasis and shorter OS and DFS (55, 56) MCT1 expression associated with shorter DFS (56)
Bladder	↑MCT1 (57) ↑ MCT4 (57)	MCT1 expression associated with advanced TNM stage and poor OS MCT4 expression associated with poor RFS (58)
Prostate	↓MCT1 (59) ↑ MCT4 (59)	MCT1 and MCT4 expression associated with advanced TNM stage (59)
Kidney	↑MCT1 (60) ↑ MCT4 (60)	MCT1 expression associated with larger tumor size and advanced TNM stage, shorter PFS (60) and OS (61), and high Fuhrman grade (62, 63) MCT4 expression correlated with reduced OS and PFS (61)
Ovarian	↑MCT1 (64) ↑ MCT4 (64)	MCT1/CD147 and MCT4 expression associated with TMN stage (64)
Cervix	↑MCT1 (65) ↑ MCT4 (65)	MCT1/CD147 expression associated with lymph-node and/or distant metastases (66)
Skin	↑MCT1 (67) ↑ MCT4 (67)	MCT1 and MCT4 expression associated with shorter OS (68)
Adrenal	+ MCT1 (69) ↑ MCT4 (69)	MCT1 expression associated with advanced TMN stage, presence of metastasis, shorter OS and DFS (69)
Hematological	↑ MCT1 (70)	MCT1 and MCT4 expression associated with high grade (71)

*↑, increased; ↓, decreased; +, positive expression; CAFs, cancer-associated fibroblasts; DFS, disease-free survival; MFS, metastasis-free survival; OS, overall survival; PFS, progression-free survival; RFS, recurrence-free survival; TNM, tumor, node, metastasis*.

## Lactate Roles in the Tumor Microenvironment: From Metabolic Affairs to Signaling

**Figure 1:**
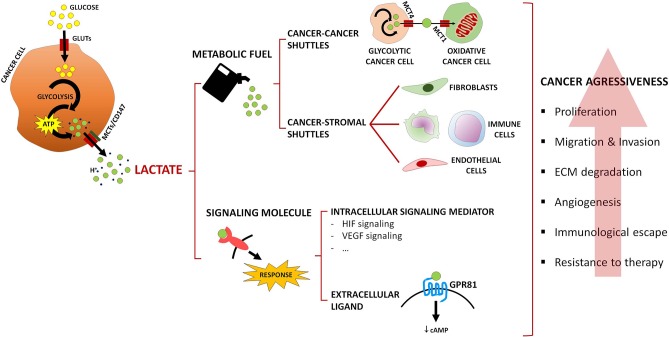
Summary of lactate roles in the tumor microenvironment. Lactate acts as a metabolic fuel, driving metabolic crosstalks involving MCT-mediated lactate shuttles among cancer cells, or between cancer cells and cancer-associated stromal cells. In addition, lactate acts as a signaling oncometabolite, intracellularly activating signaling pathways or acting as an extracellular ligand of the lactate receptor GPR81. Ultimately, cancer aggressiveness features are promoted, such as proliferation, migration, and invasion of cancer cells, extracellular matrix (ECM) degradation, angiogenesis, immunological escape, and resistance to therapy.

Tumor growth occurs under a nutrient/oxygen-restrictive microenvironment where cancer cells are enrolled in a reprogrammed metabolism that allows them to surpass those limitations while facilitating malignant dissemination. Not only cancer cells, but also cancer-associated stromal cells, take part in such scavenging program, being cytosolic lactate the main driver of those metabolic alterations. Lactate is formed exclusively from pyruvate regardless of oxygen availability, and robustly exported to the TME, reaching concentrations that can be 20-fold higher (about 40 mM) (74) than in non-tumoral tissues (about 1.5–3 mM) (13, 15). Lactate is a major fuel source, providing energetic and anabolic support to cancer cells, and an important oncometabolite with both extracellular and intracellular signaling functions that equally contribute to cancer progression (Figure 1) (75, 76).

### Lactate as a Metabolic Substrate

Lactate acts as a powerful regulator of multiple hallmarks of cancer, supporting cell proliferation and promoting immune suppression, angiogenesis, migration, metastasis, and resistance to therapy (20), namely chemotherapy (77), radiotherapy (78), and targeted therapy (79, 80). Cancer cells exploiting aerobic glycolysis upregulate GLUTs and MCTs, secreting large amounts of lactate (Figure 1), while deviating glycolytic intermediates to fuel alternative anabolic pathways (e.g., pentose phosphate pathway), thus sustaining rapid cell proliferation (81). Due to the metabolic heterogeneity of the TME, cancer cells are also able to engage into context-dependent metabolic affairs, similarly to what occurs in muscle (82) and brain (83). One such example is the symbiosis between well and poorly-oxygenated cancer cell populations (Figure 1): at the hypoxic, nutrient-poor/normoxic, nutrient-rich interface, lactate is released by glycolytic cancer cells through MCT4, and taken up by oxidative cancer cells through MCT1, where it fuels oxidative phosphorylation, thus sparing glucose for glycolytic cancer cells (84). Occurrence of this “two compartment model” was additionally described between lactate-avid breast cancer cells and “corruptible” glycolytic cancer-associated fibroblasts (CAF) by Lisantis's group (85), and further amplified to a “three compartment model” in the study by Curry et al. (86); in head and neck cancer samples, the authors showed that catabolic compartments composed of Warburg-adapted MCT4-expressing cancer cells and CAFs provided anabolic MCT1-expressing cancer cells with glycolysis-originating lactate (86). Since those original observations, numerous studies reported similar associations in several cancer models, such as non-Hodgkin lymphoma (87), pancreas (88), lung (89), prostate (90), and bladder (91); clinically, this metabolic phenotype has been associated with cancer aggressiveness, resistance to therapy and poor survival (89–91). Several mechanisms have been pointed out as mediators of those metabolic affairs, such as secretion of growth factors [e.g., cancer cell-secreted basic fibroblast growth factor (bFGF) in response to CAF-secreted hepatocyte growth factor (HGF) (92)], interleukins [e.g., IL-1β secretion by cancer cells (93)], and exosomal microRNAs (94). Interestingly, microRNA-containing exosomes secreted by CAFs were able to inhibit oxidative metabolism in cancer cells, while providing them with intact metabolites, namely glucose, to sustain their growth (95). In such an inverted scenario, CAFs oxidize cancer cell-derived lactate to support tumor proliferation (96); this has been correlated with resistance to targeted therapy (80).

In addition to CAFs, the metabolic promiscuity described above involves other cells of the TME, namely immune and endothelial cells (Figure 1). Cytotoxic T cells' transition from an anergic to a fully activated state relies on an accelerated glucose metabolism (97) and, in a glucose-restricted TME, cancer cells easily succeed in such metabolic competition (98). Dampening of lymphocyte proliferation and motility, cytokine production and cytotoxic activity ultimately leads to immunosuppression, as a result of the excess cancer cell-derived lactate that blocks lactate export by immune cells (99) and might be inclusively taken up by those cells, thus impairing their glycolysis-dependent activation (100). Lactate also mediates polarization of macrophages from an M1- (anti-tumoral type) to an M2-like phenotype (pro-tumoral type) (101, 102); induction of VEGF (vascular endothelial growth factor) expression has been linked to this pro-tumoral state (103). Moreover, it was proposed that endothelial cells rely on extracellular lactate uptake, via MCT1, as a fuel source for their oxidative metabolism, promoting VEGF/VEGFR-2 production through HIF-1α stabilization, endothelial cell migration and tube formation (104–106).

Lactate stimulates motility, migration and invasion of cancer cells (38, 107), as a probable result of CD44 expression and hyaluronan production (108), as well as activation of matrix metalloproteinases (MMPs) (109), both promoted by extracellular acidosis.

### Lactate as a Signaling Metabolite

As stated above, lactate can serve additional purposes beyond acting as a metabolic substrate, functioning as an intracellular signaling mediator and as an extracellular ligand. At the intracellular level (Figure 1), hypoxia adaptation is assured by lactate in HIF-1α-dependent [direct HIF-1α stabilization by prolyl hydroxylase (PHD) 2 inhibition (110)] and independent [binding to N-Myc downstream-regulated (NDRG3) protein, preventing association with PHD2 (111)] fashions. HIF-2α stabilization is also induced by lactate, which ultimately potentiates glutaminolysis in cancer cells (112). Lactate promotes HIF-1α-mediated VEGF expression in the cancer cell, and expression of bFGF and VEGFR-2 by neighboring endothelial cells (105). Apart from this HIF-1α-dependent angiogenic signals, endothelial cells are also activated by NF-κB stabilization (104) in a lactate-dependent manner. NF-κB activation in cancer cells' instructed CAFs additionally drives resistance to targeted therapies, being lactate secreted by cancer cells the instructor in such phenotype (80). Pyruvate kinase M2/HIF-1α-driven gene expression in prostate cancer cells promoted epithelial-to-mesenchymal in response to CAF-secreted lactate (113). Immune suppression is also mediated by lactate signaling, as different studies reported that this oncometabolite interferes with key tumor pathways that lead to IFN-γ production by cytotoxic T cells (114), activates the IL-23/IL-17 proinflammatory pathway (115) and promotes polarization of macrophages toward an M2-like phenotype (103). Recently, functions in histone post-translational modification, termed histone lysine lactylation, were attributed to lactate and shown to regulate gene expression in macrophages; increased lactate production led to this epigenetic modification, inducing an M2-like phenotype during wound healing (116).

The signaling functions of lactate at the extracellular space (Figure 1) are mediated by the lactate-activated G-protein-coupled receptor GPR81 (5, 117). Its expression is not limited to the plasma membrane but also to other intracellular organelles (118). GPR81 activation occurs at a lactate concentration of 0.2–1.0 mM (119), followed by cyclic AMP downregulation and inhibition of protein kinase A (PKA)-mediated signaling (120). In the physiological context, lactate binds to GPR81, which inhibits lipolysis in fat cells (121). In the cancer context, modulation of lactate-sensing proteins, such as MCTs, ultimately leads to tumor proliferation and dissemination (122), escape from the immune system (123) and therapy resistance (124). GPR81 expression is upregulated in cervical (125), breast (126) and liver cancer (122), and associated with the progression of cervical squamous carcinoma (125). *GPR81* is highly expressed in different cancer cell lines including colon, breast, lung, hepatocellular, cervical, and pancreatic (122, 126). *In vitro*, GPR81 expression associated with cancer cell survival, proliferation, migration, invasion and resistance to chemotherapy, and is involved in the suppression of antitumor immunity by promoting the overexpression of PD-L1 in lung cancer cells lines (123, 124, 126). Knockdown of GPR81 in a xenograft cancer model resulted in reduction of tumor growth and metastasis (122, 126).

The putative lactate sensors GPR4, GPR65, GPR68, and GPR132 have been described as proton-sensitive, and are activated at the acidic TME due to the low pH levels obtained from lactic and carbonic acids (5). GPR132 and GPR65 were additionally described in macrophages and linked to their polarization toward a pro-tumoral phenotype (127, 128). It remains to be clarified whether the modulation of lactate-sensing signaling pathways occurs through a direct GPR-lactate interaction ou through a conformational modification in the receptor induced by lactic acidosis.

## Lactate Metabolism as A Prognostic and Therapeutic Tool

As mentioned above, lactate levels in tissues can mirror their metabolic status. Lactate concentrations vary either within healthy or diseased tissues, reflecting the distribution of the metabolic activity in the tissue, phenomenon known as “metabolic zonation” (129). This term was first described in the liver, in which there is hepatocyte metabolic heterogeneity along the porto-central axis, resulting from the physiological occurring oxygen gradient (130). In solid malignant tumors, which are characterized by high heterogeneity, “metabolic zonation” results from different intrinsic properties of cancer cells, co-existence of different cell populations within the tumor and different distribution of the vascular supply (129). The clinical significance of the variable levels of lactate in human solid tumors was first described in 2000 by the group of Walenta et al. (131), where significantly higher lactate levels in cervical metastatic tumors were found, compared with non-metastatic malignancies, suggesting that tumor lactate content could be used as a prognostic biomarker. Interestingly, the levels of lactate were inversely correlated with the levels of glucose, and directly correlated with the expression of MCT4. Later studies have also linked intratumoral lactate levels with higher incidence of distant metastasis and poor patient survival (76).

Prognostic value has also been attributed to important players in lactate metabolism, namely involved in lactate production (LDHA) and lactate transport (MCTs). There are several studies on the prognostic value of LDH levels, supported by systematic reviews/meta-analyses (132). As examples, higher pretreatment LDH concentration is associated with increased risk of overall mortality in lung cancer patients (133), high LDH serum levels are associated with lower event-free survival (EFS) in osteosarcoma patients (134) and with overall survival (OS)/progression-free survival (PFS) in urinary system cancer patients (135). Besides serum LDH, LDHA levels in cancer tissues have been reported as a biomarker of malignancy and prognosis (136). As examples, upregulation of LDHA levels in pancreatic and esophageal cancer have been associated with metastasis, tumor stage, tumor recurrence, and patient survival (137). However, LDHA expression in malignant tumor tissues does not correlate consistently with serum LDH levels, which may indicate that these are independent prognostic factors in cancer (138, 139). Studies on lactate transporters (MCTs/CD147) are not as solid as for LDH, but also reveal prognostic value (Table 1) (41). In a recent meta-analysis Bovenzi et al. (140) identified association between increased MCT4/CD147 expression with decreased OS and disease-free survival (DFS) across many cancer types, while there was no clear association for MCT1 expression with these parameters.

Besides prognostic biomarkers, both LDHA and MCTs have been recognized as attractive targets for cancer therapy. LDHA overexpression has been associated with increased cancer aggressiveness and targeting has been tackled both genetically and pharmacologically. There are different types of LDHA inhibitors, including the pyruvate-competitive (e.g., oxamate), the NADH-competitive (e.g., gossypol), the pyruvate and NADH-competitive (N-hydroxyindoles), and the free enzyme-binding inhibitors (galloflavin) (138). LDH pharmacological inhibition reduces lactate production, impairs cell proliferation *in vitro*, and reduces tumor size *in vivo*, either with LDH inhibitors alone or in combination with other agents (138, 141). Additionally, gossypol has also demonstrated promising results in different clinical trials, being relatively safe and effective in reducing tumor markers (138, 142). These results are supported by LDHA silencing studies in tumor models, where cell proliferation, migration and tumor growth were prevented (143, 144). However, genetic studies deleting LDHA/LDHB or glucose phospho-isomerase (GPI) have demonstrated that Warburg effect is dispensable as agressive tumors, relying on OXPHOS, are able to survive and to develop tumors in nude mice (145).

As stated above, upregulation of MCT1 and MCT4 has been described in a variety of human cancers, and inhibition of MCT activity has been showing promising results in pre-clinical models (41). *In vitro*, MCT inhibition impairs lactate transport, cell proliferation, invasion and migration, and induces cell death, while it delays tumor growth, induces necrosis and decreases invasion *in vivo*. MCT activity has been inhibited either genetically (gene downregulation or knockout) or using pharmacological inhibitors. MCT classical inhibitors include the α-cyano-4-hydroxycinnamate CHC, 4,4′-di-isothiocyanostilbene-2,2′-disulfonates (e.g., DIDS) and flavonoids (e.g., quercetin) (15). However, these inhibitors are not MCT/MCT isoform specific, which prompted the search for new inhibitors. AstraZeneca developed MCT1 specific inhibitors, and one of them (AZD3965) already reached clinical trials in the cancer setting. After a first evaluation of compound tolerability, the trial is now set to evaluate the effect of AZD3965 in MCT1 positive tumors with pre-clinical positive results (146).

Information on the use of lactate by tumors could also be of value in cancer therapy. Van Hée et al. developed a PET tracer of lactate [(±)- [^18^F]-3-fluoro-2-hydroxypropionate, [^18^F]-FLac] to monitor MCT1-dependent lactate uptake in tumors (147). The authors propose that this tracer can be used to predict response to treatments that disrupt lactate consumption, with potential to allow personalized patient treatment.

## Discussion

Along the previous decades, the role of lactate has been overlooked, as it was seen as a mere metabolic waste of cell glycolytic metabolism. However, recent evidence has been revealing new and important oncogenic roles of lactate in malignant tumors. Lactate can function either as metabolic fuel for oxidative cells or as signaling molecule in the TME, being responsible for several aggressiveness cancer cell features, namely proliferation, migration and invasion, angiogenesis, escape to the immune system and resistance to therapy (Figure 1). Besides, upregulation of key proteins involved in lactate metabolism, namely LDHA and MCTs, have demonstrated clinical prognostic value and are seen as rational targets for cancer therapy. Thus, given the important role of lactate metabolism in cancer aggressiveness and response to therapy, lactate metabolism inhibitors should to be further explored in the clinical setting, especially in combination with classical therapy, molecular targeted drugs and immunotherapy.

## Author Contributions

All authors listed have made a substantial, direct and intellectual contribution to the work, and approved it for publication.

### Conflict of Interest

The authors declare that the research was conducted in the absence of any commercial or financial relationships that could be construed as a potential conflict of interest. The handling editor declared a shared affiliation, though no other collaboration, with one of the authors FB.
